# Systematic Investigation of the Reliability of the Frozen Nuclei Approximation for Short-Pulse Excitation: The Example of HCCI^+^

**DOI:** 10.3389/fchem.2022.857348

**Published:** 2022-03-16

**Authors:** Dongming Jia, Yonggang Yang

**Affiliations:** ^1^ MOE Key Laboratory for Non-equilibrium Synthesis and Modulation of Condensed Matter, School of Physics, Xi’an Jiaotong University, Xi’an, China; ^2^ State Key Laboratory of Quantum Optics and Quantum Optics Devices, Institute of Laser Spectroscopy, Shanxi University, Taiyuan, China; ^3^ Collaborative Innovation Center of Extreme Optics, Shanxi University, Taiyuan, China

**Keywords:** frozen nuclei approximation, ultrashort laser pulses, nuclear quantum dynamics, electronic excitation, population transfer

## Abstract

In this work we quantitatively study the reliability of the frozen nuclei approximation for ultrafast dynamics. Specifically we study laser excitation of HCCI^+^ from its ground state to the first electronically excited state. The population of the first excited state is obtained by both the frozen nuclei approximation and by multidimensional nuclear dynamics. Detailed comparison of the results by the two methods are performed to provide quantitative criteria for the reliability of the frozen nuclei approximation for this system.

## 1 Introduction

The rapid advances of ultrafast science and technology have made it possible to manipulate electron dynamics in molecular systems with ultrashort laser pulses. In particular, laser induced electron density redistribution such as charge transfer (Marcus, 1956; May and Kühn, 2008) and charge migration (Weinkauf et al., 1996, 1997; Cederbaum and Zobeley, 1999; Calegari et al., 2014; Kraus et al., 2015) have been extensively investigated. In general, charge migration prefers frozen nuclei or small amplitude nuclear motions, while charge transfer is typically accompanied by large amplitude nuclear motions. The research of charge transfer processes has a relatively long history. While ultrafast charge migration emerged as a hot topic during the past two decades (Weinkauf et al., 1996, 1997; Remacle et al., 1998; Cederbaum and Zobeley, 1999; Remacle and Levine, 1999; Barth and Manz, 2006; Kanno et al., 2006; Yudin et al., 2006; Remacle et al., 2007; Kanno et al., 2010; Mineo et al., 2012; Calegari et al., 2014; Kraus et al., 2015; Li et al., 2015; Yamaki et al., 2016; Wörner et al., 2017; Mineo et al., 2021). It should be noted that the first attosecond charge migration phenomenon was already introduced in 1944 (Eyring et al., 1944) and was largely forgotten during the next decades. Surveys of the literature on ultrafast charge migration can be found in Ref. (Jia et al., 2017a; Wörner et al., 2017). Below we summarize some typical features of ultrafast charge migration and its connection to the frozen nuclei approximation (FNA).

Ultrafast charge migration typically represents quantum dynamics of a coherent superposition of more than one electronic state. The typical time scale of ultrafast charge migration ranges from several hundred attoseconds to a few femtoseconds which makes the experimental observation (Kraus et al., 2015) rather difficult. For such a short time, the frozen nuclei approximation has been widely used for theoretical work of ultrafast charge migration. There are also several theoretical investigations which include the effects of nuclear motions (Bandrauk et al., 2009; Kanno et al., 2010; Ulusoy and Nest, 2012; Mendive-Tapia et al., 2013; Mineo et al., 2014; Despré et al., 2015; Mineo et al., 2021). The amplitude of charge migration can be significantly modulated by nuclear motions, in particular for relatively long-time dynamics (Mendive-Tapia et al., 2013; Mineo et al., 2014; Jia et al., 2019a,b). In general, the FNA is widely believed to be only valid for short time pulses, but there are no quantitative criteria for how short the pulses should be. This serves as the motivation for the present work: to seek quantitative criteria for the reliability of the FNA. Specifically, we will investigate short-pulse excitations of HCCI^+^ by systematically varying the laser parameters in a sufficiently wide region.

The choice of HCCI^+^ as our model of interest is based on the availability of experimental data (Heilbronner et al., 1971; Kraus et al., 2015) and theoretical techniques (Jia et al., 2019b). The combined experimental and theoretical reconstruction of attosecond charge migration has been reported for ultrafast ionization of HCCI (Kraus et al., 2015). Coherent superposition of the ground and first excited states has been created and analyzed. Subsequent theoretical investigations of ultrafast charge migration in HCCI^+^ (Jia et al., 2017b; Ding et al., 2017) related to the experimental observation (Kraus et al., 2015) exploit the FNA. In-depth investigations of simulations and manipulations of charge migration in HCCI^+^ including multidimensional nuclear dynamics have been reported recently (Jia et al., 2019a,b). However, no comparisons between the results of multidimensional nuclear dynamics and the ones of the FNA are available.

In the present work, we will investigate the reliability of the FNA by comparing the FNA and multidimensional nuclear dynamics. The remainder parts of the paper are organized as follows. Section 2 contains the model and methods for numerical calculations. Section 3 presents the results and discussion. The conclusions are drawn in Section 4.

## 2 Model and Methods

We focus on laser excitations of HCCI^+^ from its ground state. Full dimensional simulations of the system involve sets of electronic coordinates ***r*** = {***r***_1_, ***r***_2_, … } and nuclear coordinates ***R*** = {***R***_1_, ***R***_2_, … }. Here ***r***_*i*_ and ***R***_*j*_ are the spatial coordinates of the *i*-th electron and the *j*-th nucleus, respectively. It is convenient to use the Dirac notation for the electronic degrees of freedom. The total wavefunction of the system is thus

$$\Psi \left(r,R,t\right)\;=\;\left\langle r|\Psi \left(R,t\right)\right\rangle .$$ (1)

Using the Born-Huang expansion (Born and Oppenheimer, 1927; Born and Huang, 1954), the total wavefunction can be expressed in terms of the electronic eigenstates |*k*(***R***)⟩ which are the solutions of the standard time-independent electronic schrödinger equation

$$H_{\text{el}}\left(R\right)|k\left(R\right)\rangle \;=\;V_{k}\left(R\right)|k\left(R\right)\rangle .$$ (2)

The corresponding electronic eigenenergy *V*_*k*_(***R***) is the *k*-th potential energy surface (PES). Here *V*_*k*_(***R***) and ⟨ ***r*** |*k*(***R***)⟩ are the same as the ones used in Refs. (Jia et al., 2019a,b) which are calculated by Molpro (Werner et al., 2012) using the state-averaged CASSCF(15,13) with cc-pVQZ basis set (cc-pVQZ-pp for iodine).

According to (Kraus et al., 2015; Jia et al., 2019a,b), nonadiabatic couplings between different electronic states |*k*(***R***)⟩ are negligible. The total Hamiltonian for HCCI^+^ in an external laser field ***E*** (*t*) can be approximated as

$$H\left(t\right)\;=\;\underset{kk^{\prime }}{\sum }|k\left(R\right)\rangle \left[T\left(R\right)\delta_{kk^{\prime }}+V_{k}\left(R\right)\delta_{kk^{\prime }}-\mu_{kk^{\prime }}\left(R\right)\cdot E\left(t\right)\right]\langle k^{\prime }\left(R\right)|,$$ (3)

where *T*(***R***) is the nuclear kinetic energy and ***μ***_*kk*′_(***R***) = ⟨*k*(***R***)| ***μ*** |*k*′(***R***)⟩ is the transition (or permanent) dipole moment. The laser pulse has a Gaussian shape with maximum amplitude *E*_max_ and carrier frequency *ω*

$$\begin{array}{c} E\left(t\right)\;=\;e_{z}\;E_{\text{max}}s\left(t\right)\sin\left(\omega t\right),\\ s\left(t\right)\;=\;e^{-at^{2}/T^{2}}, \end{array}$$ (4)

where ***e***_*z*_ is the direction of the electric field. For convenience, the electric field and the molecules are oriented along the *z*-axis. In the literature, there are different choices of the parameter *a* in Eq. 4. For the present work we set

a=4⁡ln⁡2 (5)

for easier characterization of the pulse duration. We define the pulse duration as the full width at half maximum (FWHM) of *s*(*t*), which is just *T* in Eq. 4.

The quantum dynamics of the system can be simulated by the time-dependent schrödinger equation subject to initial condition at *t* = −*∞*

$$\begin{array}{cc} i\hbar \frac{d}{dt}|\Psi \left(R,t\right)\rangle \; & =\;H|\Psi \left(R,t\right)\rangle ,\\ |\Psi \left(R,t=-\infty \right)\rangle \; & =\;\chi_{g,v=0}\left(R\right)|g\left(R\right)\rangle , \end{array}$$ (6)

where *χ*_*g*,*v*=0_(***R***) is the vibrational ground state wavefunction of the lowest potential energy surface *V*_*g*_(***R***). For convenience we use *k* = *g*, *e* to represent the lowest and first excited electronic states, respectively. The wave packet is numerically propagated by means of the split operator method (Leforestier et al., 1991).

Subsequently, we can obtain the population of the electronic state |*k*(***R***)⟩ according to

$$\begin{array}{cc} P_{k}\left(t\right)\; & =\;\int \langle \Psi \left(R,t\right)|k\left(R\right)\rangle \langle k\left(R\right)|\Psi \left(R,t\right)\rangle dR\\ & \equiv \int \chi_{k}^{*}\left(R,t\right)\chi_{k}\left(R,t\right)dR, \end{array}$$ (7)

where *χ*_*k*_ (***R,t***) = ⟨*k*(***R***)|Ψ(***R***, *t*)⟩ is the nuclear wave packet on the *k*-th PES *V*_*k*_(***R***). It contains seven vibrational coordinates. According to Ref. (Jia et al., 2019a,b), one-dimensional (1D), three-dimensional (3D), and seven-dimensional (7D) calculations lead to essentially the same results. In the 3D calculations, the H-C, C-C and C-I bond lengths are explicitly taken into account and the four bending degrees of freedom are neglected. This kind of approximation is reasonable for linear molecules, such as HCCI^+^. In the present work we use the same 3D calculations for the nuclear wave packet *χ*_*k*_ (***R,t***) as in Ref. (Jia et al., 2019b). Then we mainly focus on the population of the first electronically excited state *P*_*k*_(*t*) for *k* = *e*.

To check the reliability of the FNA, we further calculated the population of the first electronically excited state $P_{e}^{\text{FNA}}$ using the FNA. Accordingly, the molecular structure is fixed at the minimum of the lowest PES *V*_*g*_(***R***). This structure is called equilibrium structure ***R***_eq_. The corresponding transition dipole moment is ***μ***_eq_ ≡ ***μ***_*ge*_ (***R*** = ***R***_eq_). The electronic wavefunction of the FNA is expanded as

$$|\Psi^{\text{FNA}}\left(t\right)\rangle \;=\;\underset{k}{\sum }c_{k}\left(t\right)|k\left(R=R_{\text{eq}}\right)\rangle .$$ (8)

the time dependent coefficient *c*_*k*_(*t*) can be obtained subject to the initial condition *c*_*k*_(*t* = −*∞*) = *δ*_*kg*_. The corresponding population is

$$P_{k}^{\text{FNA}}\left(t\right)\;=\;|c_{k}\left(t\right){|}^{2}.$$ (9)

Throughout this work we fix the carrier frequency of the laser in Eq. 4 as *ℏω* = *V*_*e*_ (***R***_eq_) − *V*_*g*_ (***R***_eq_). We only focus on the final population at *t* = *t*_*f*_ when the laser pulse is off. This leads to the following analytical expression (Jia et al., 2017a)

$$P_{e}^{\text{FNA}}\left(t=t_{f}\right)\approx \sin^{2}\left(\frac{\sqrt{\pi }E_{\text{max}}\mu_{\text{eq}}^{z}T}{\sqrt{a}\hbar }\right),$$ (10)

where $\mu_{\text{eq}}^{z}$ denotes the *z* component of the transition dipole at ***R***_eq_. The relative error of the FNA with respect to multi-dimensional nuclear dynamic is defined as

$$\frac{\delta P_{e}}{P_{e}}\;=\;\frac{\left|P_{e}^{\text{FNA}}\left(t=t_{f}\right)-P_{e}\left(t=t_{f}\right)\right|}{P_{e}\left(t=t_{f}\right)}\times 100\%.$$ (11)

For all the subsequent numerical calculations we set *t*_*f*_ = 5*T*. However, this should not be wrongly interpreted as the FNA is valid even for *t* = 5*T*. We choose *t*_*f*_ = 5*T* just to make use of the property that the results presented in this work do not depend on different choices of *t*_*f*_ as long as *t*_*f*_ ≥ *T*.

## 3 Results and Discussion

**FIGURE 1:**
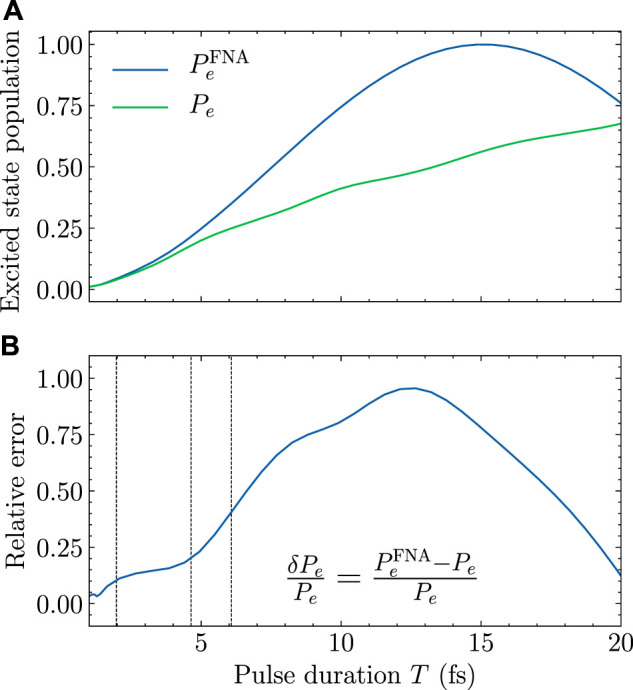
Population of the first electronically excited state of HCCI^+^ versus the pulse duration *T*, at *t* = *t*_*f*_ when the laser pulse is switched off. The maximum-amplitude of the electric field *E*_max_ is 2 GV/m. **(A)** *P*_*e*_ (*t*_*f*_) and $P_{e}^{\text{FNA}}\left(t_{f}\right)$ obtained by multidimensional nuclear dynamics (green) and by the frozen nuclei approximation (blue), respectively. **(B)** The relative error $\frac{\delta P_{e}}{P_{e}}$ of $P_{e}^{\text{FNA}}\left(t_{f}\right)$ with respect to *P*_*e*_ (*t*_*f*_). Vertical lines show the positions of $\frac{\delta P_{e}}{P_{e}}\;=\;10\%$ , 20%, and 40%, respectively.

The equilibrium structure ***R***_eq_ of HCCI^+^ is linear with bond lengths *R*_HC_ = 1.06 Å, *R*_CC_ = 1.21 Å and *R*_CI_ = 1.95 Å. The corresponding vertical excitation energy from ground state |*g*(***R***_eq_)⟩ to the first excited state |*e*(***R***_eq_)⟩ is *ℏω* = 2.41 eV. For typical pulse durations, there are sufficient numbers of cycles in ***E*** (*t*) to make the electronic transition resonant. The corresponding transition dipole has only a *z*-component, which is $\mu_{\text{eq}}^{z}\;=$ 3.21 Debye. Subsequently we calculated the population of the first electronically excited state $P_{e}^{\text{FNA}}\left(t=t_{f}\right)$ according to Eq. 10 by the FNA. Convergence tests are performed for nuclear dynamics simulations such that the corresponding population of the first electronically excited state *P*_*e*_(*t*) does not change subject to further increase of the grid-region or decrease of the spatial or temporal steps. We first analyze the dependence of the results on the pulse durations with the other parameters fixed. Specifically, the maximum amplitude of the electric field *E*_max_ is fixed at 2.0 × 10^9^ V/m. The detailed comparison between *P*_*e*_(*t* = *t*_*f*_) and $P_{e}^{\text{FNA}}\left(t=t_{f}\right)$ is shown in Figure 1 for *T* ≤ 20 fs.

As can be seen from Figure 1A, the deviation between $P_{e}^{\text{FNA}}\left(t_{f}\right)$ and *P*_*e*_(*t*_*f*_) gradually increases with the pulse duration *T*, for the region of short pulses. For *T* ≤ 20 fs, *P*_*e*_(*t*) keeps increasing with *T*. However, $P_{e}^{\text{FNA}}\left(t\right)$ first increases and then decreases for *T* ≤ 20 fs. This kind of qualitative deviation will be further discussed below. According to Eq. 10 $P_{e}^{\text{FNA}}\left(t_{f}\right)$ will oscillate periodically with the pulse duration *T*. In Figure 1A, $P_{E}^{\text{FNA}}\left(t_{f}\right)$ reaches its maximum $P_{e,max}^{\text{FNA}}\;=\;1$ for *T* = 15.45 fs. However, *P*_*e*_(*t*_*f*_) is still substantially below one even for *T* = 20 fs.

To quantitatively compare $P_{e}^{\text{FNA}}\left(t_{f}\right)$ and *P*_*e*_(*t*_*f*_), the relative error $\frac{\delta P_{e}}{p_{e}}$ defined in Eq. 11 is shown in Figure 1B. The relative error increases relatively slowly when the pulse duration *T* is smaller than 5 fs, and increases rapidly when *T* is larger than 5 fs. For long pulses, say *T* ≥ 15 fs, Figure 1B shows significant decrease of $\frac{\delta P_{e}}{p_{e}}$ . However, this is pure coincidence. As can be identified from Figure 1A, the trend of $P_{e}^{\text{FNA}}\left(t_{f}\right)$ is already qualitatively wrong for *T* > 15.45 fs. Smaller relative error in this region does not imply better agreement between the frozen nuclei approximation and real physics. Consequently, we focus on short pulses for which the FNA is expected to be reasonable. Accordingly, we add three vertical lines in Figure 1B for relative errors of 40, 20, and 10%, respectively. The corresponding pulse durations with fixed value of *E*_max_ = 2.0×10^9^ V/m are *T* = 6.09, 4.65, and 1.97 fs, respectively.

**FIGURE 2:**
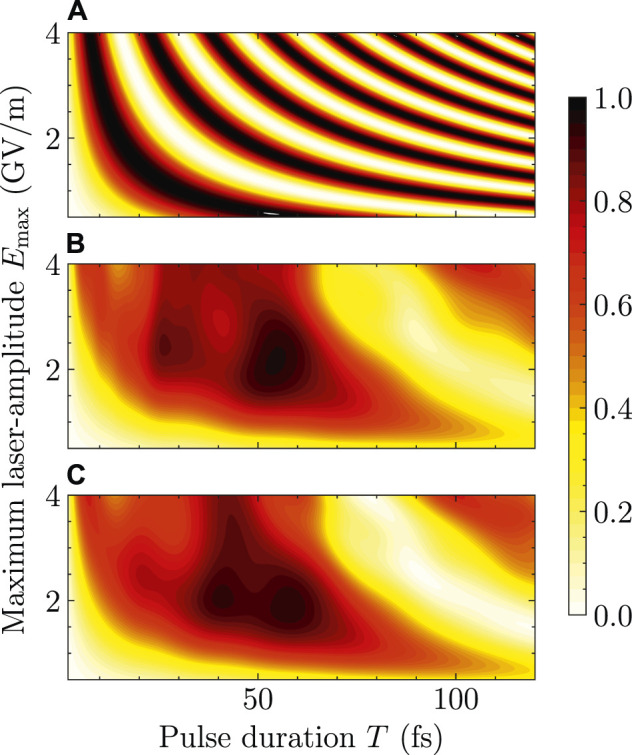
Population of the first electronically excited state of HCCI^+^ *versus* *E*_max_ and *T* illustrated by color-coded contour plots. **(A)** $P_{e}^{\text{FNA}}\left(t_{f}\right)$ calculated by the FNA. **(B)** *P*_*e*_ (*t*_*f*_) obtained by 3D nuclear dynamics simulations. **(C)** Same as **(B)** but the nuclear dynamics is one-dimensional.

Figure 2 shows the color-coded contour plots for the dependence of $P_{e}^{\text{FNA}}\left(t_{f}\right)$ and *P*_*e*_ (*t*_*f*_) on the amplitude of the electric field *E*_max_ and the pulse duration *T*. The full set of the involved parameters span the region 0.5 GV/m ≤ *E*_max_ ≤ 4.0 GV/m and *T* ≤ 120 fs. The region of *E*_max_ more or less covers the reported amplitudes of lasers exploited in typical applications in the literature. The region of *T* reaches the first revival of charge migration in HCCI^+^ reported in Ref. (Jia et al., 2019b). As can be seen from Figure 2A, $P_{e}^{\text{FNA}}\left(t_{f}\right)$ oscillates between 1 and 0 periodically with *E*_max_ or *T*. Larger values of *E*_max_ or *T* corresponds to smaller oscillation period of $P_{e}^{\text{FNA}}\left(t_{f}\right)$ , c.f., Eq. 10. Figure 2B is the same as Figure 2A except that $P_{e}^{\text{FNA}}\left(t_{f}\right)$ is replaced by *P*_*e*_(*t*_*f*_) which is obtained by performing 3D nuclear dynamics simulations. We can immediately identify that Figures 2A,B are qualitatively different for relatively long laser lulses (e.g., for *T* ≥ 20 fs). We therefore consider the results for the FNA are not meaningful for relatively long laser pulses. This is quite natural. Even if $P_{e}^{\text{FNA}}\left(t_{f}\right)$ is coincidentally very close to *P*_*e*_(*t*_*f*_) for *T* ≥ 20 fs, the nuclear wave packet is quite different from the initial one as has been reported in Ref. (Jia et al., 2019b). Accordingly, the basic assumption of the FNA breaks down for *T* ≥ 20 fs. We further plot Figure 2C which is the same with Figure 2B except that the population of the first electronically excited state is obtained by performing 1D nuclear dynamics simulations which explicitly treats the C-I stretch (Jia et al., 2019a). The results of 1D and 3D simulations agree well with each other, which confirms the findings in Refs. (Jia et al., 2019a,b).

**FIGURE 3:**
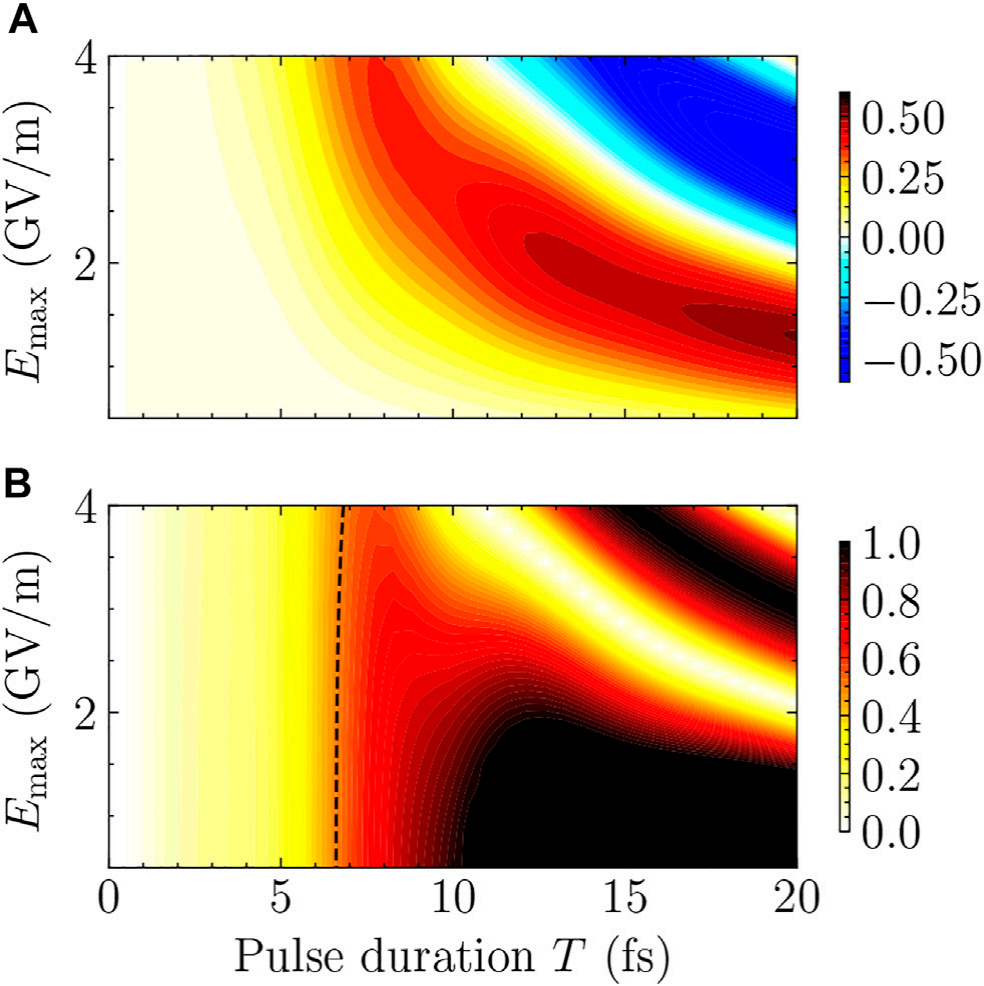
**(A)** Color-coded contour plots for the deviation $P_{e}^{\text{FNA}}\left(t_{f}\right)-P_{e}\left(t_{f}\right)$ between $P_{e}^{\text{FNA}}\left(t_{f}\right)$ in Panel 2A and *P*_*e*_ (*t*_*f*_) in Panel 2B for *T* ≤20 fs. **(B)** Color-coded contour plots for the relative error $\frac{\delta P_{e}}{P_{e}}$ of $P_{e}^{\text{FNA}}\left(t_{f}\right)$ with respect to *P*_*e*_ (*t*_*f*_). The black dashed curve indicates $\frac{\delta P_{e}}{P_{e}}=50\%$ .

To systematically study the reliability of the FNA for short pulses, the difference between $P_{e}^{\text{FNA}}\left(t_{f}\right)$ in Figure 2A and *P*_*e*_ (*t*_*f*_) in Figure 2B is shown in Figure 3A, for the region *T* ≤ 20 fs when the FNA may be expected to work. The dependence of $P_{e}^{\text{FNA}}\left(t_{f}\right)-P_{e}\left(t_{f}\right)$ on *E*_max_ and *T* is shown by color-code contour plots. Results for which $|P_{e}^{\text{FNA}}\left(t_{f}\right)-P_{e}\left(t_{f}\right)|\geq 0.5$ are not resolved, since results with errors larger than 50 percent are in general not helpful. For any fixed value of *E*_max_, the deviation between $P_{e}^{\text{FNA}}\left(t_{f}\right)$ and *P*_*e*_ (*t*_*f*_) first increases then decreases with *T* after reaching a maximum. More complicated features can be found for relatively large *E*_max_ combined with relatively long *T*. However, as discussed above for Figure 1, the results of $P_{e}^{\text{FNA}}\left(t_{f}\right)$ in this complicated region in Figure 3A (specifically, after reaching maximum deviations) can agree better with *P*_*e*_(*t*_*f*_) by coincidence. In the following we only focus on the left bottom region of Figure 3A before the corresponding deviation $P_{e}^{\text{FNA}}\left(t_{f}\right)-P_{e}\left(t_{f}\right)$ reaches its maximum for any fixed value of *E*_max_. As can be seen from Figure 3A, the deviations are rather small for *T* ≤ 5 fs for all the different values of *E*_max_ involved in the present work. This implies that we may roughly use *T* ≤ 5 fs as the criterion for the reliability of the FNA. The deviation not only increases with *T* for any given *E*_max_, but also increases with *E*_max_ for any given *T*. According to Eq. 10, $P_{e}^{\text{FNA}}\left(t_{f}\right)$ increases with the product of *E*_max_ and *T* before reaching its maximum. From Figure 3A we can also find that the deviation $P_{e}^{\text{FNA}}\left(t_{f}\right)-P_{e}\left(t_{f}\right)$ essentially increases with the product of *E*_max_ and *T* before reaching its maximum. Better criteria for the reliability of the FNA can be obtained by analyzing the relative error of $P_{e}^{\text{FNA}}\left(t_{f}\right)$ with respect to *P*_*e*_ (*t*_*f*_) shown in Figure 3B. In the same spirit, we only need to focus on the left region of Figure 3B indicated by the dashed curve in which the relative error $\frac{\delta P_{e}}{P_{e}}$ never exceeds 50%. For relatively long pulses, say *T* = 7 fs, the relative error of the FNA is already larger than 50% for any value of *E*_max_ shown in Figure 3B. In this case, the FNA is no longer reliable for *T* ≥ 7 fs.

In the short pulse region, the relative error $\frac{\delta P_{e}}{P_{e}}$ increases with the pulse duration *T*. To give quantitative criteria for the reliability of the FNA, we define certain characteristic pulse durations as follows:

$$TN:\;\frac{\delta P_{e}}{P_{e}}\leq \left(100-N\right)\%\;if\;T\leq TN.\;\;\left(N=60,80,90,95\right)$$

For example, if we want to use the FNA to obtain results with relative errors smaller than 5%, we need to set the pulse durations of the lasers to be smaller than *T*95. Similarly for the meanings of *T*90, *T*80, and *T*60. According to our model the characteristic pulse durations *T*95, *T*90, *T*80, and *T*60 only depend on one parameter *E*_max_, which will be investigated subsequently.

**FIGURE 4:**
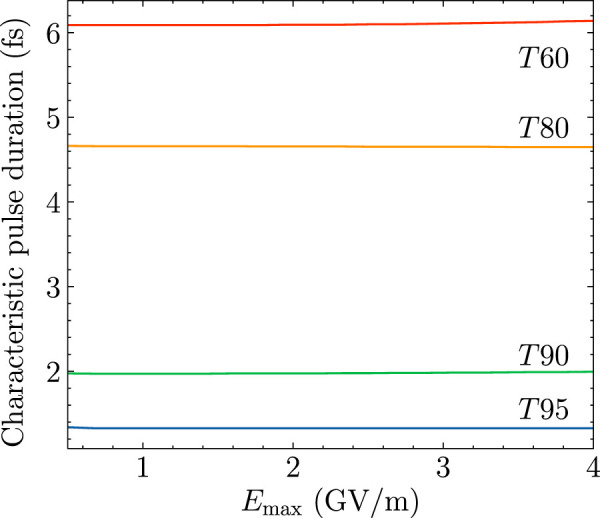
Characteristic pulse durations *T*95, *T*90, *T*80, and *T*60 for relative error $\frac{\delta P_{e}}{P_{e}}\;=$ 5, 10, 20, and 40%, respectively. See the text for more details.

The detailed dependence of *T*95, *T*90, *T*80, and *T*60 on the maximum amplitude of the electric field *E*_max_ is shown in Figure 4. A quite good property for the characteristic pulse durations is that *T*95, *T*90, and *T*80 almost do not depend on *E*_max_. The corresponding values are *T*95 = 1.32 fs, *T*90 = 1.97 fs, and *T*80 = 4.65 fs respectively. The value of *T*60 increases with *E*_max_ extremely slightly from 6.09 to 6.14 fs. For relatively high standard criteria, say relative errors below 20%, the corresponding characteristic pulse durations are quite robust with respect to different amplitudes of lasers. This greatly simplifies the criteria for choosing proper lasers for applications of short pulse excitations of HCCI^+^. Essentially, we only need to care about the durations of the laser pulses with quantitative guidance derived from Figure 4 for the reliability of the FNA.

The FNA only considers the electronic degrees of freedom and neglects the nuclear motions. Mathematically this corresponds to a large overlap of the time-dependent and the initial nuclear wave packets. The overlap can be estimated as the product of the corresponding overlap for each normal mode. The overlap for a normal mode may be approximated as $e^{-f^{2}\left(t\right)}$ for short time dynamics. Here *f*(*t*) is the displacement of the normal coordinate with respect to its initial value in units of the standard deviation of the initial wave packet for this normal mode. Typically, there will be only one or a few modes with *f* ^2^(*t*) substantially above zero, which are called active modes. The overall overlap is thus mainly determined by the active modes. For the present case, there is only one active mode which is the C-I stretch with period 86 fs (Jia et al., 2019b). Due to the relatively large amplitude of the C-I stretch mode, the function $e^{-f^{2}\left(t\right)}$ quickly decreases to zero (Jia et al., 2019a,b). In this case, the duration of the pulse must be much shorter than a vibrational period to keep the overlap large enough. According to the results of Figure 4, all the characteristic pulse durations of HCCI^+^ are smaller than $\frac{1}{10}$ of a vibrational period. However, for some molecules with sufficiently small vibrational amplitudes for all the active modes, the overlap can be relatively large for a rather long time. For such cases, the effects of nuclear dynamics can be neglected for a longer time than just a few femtoseconds (Kanno et al., 2010; Ulusoy and Nest, 2012; Despré et al., 2015).

## 4 Conclusion

We have systematically investigated the population of the first electronically excited state of HCCI^+^ excited by different laser pulses. The amplitudes and durations of the laser pulses span a rather large domain for typical applications. The deviations between the results obtained by the frozen nuclei approximation and the ones obtained by multidimensional nuclear dynamics are calculated and analyzed in detail to check the reliability of the FNA. As expected the validity of the FNA can be admitted for sufficiently short laser pulses. Quantitative criteria for the reliability of the FNA are obtained. Specifically if we want to limit the relative errors of the FNA within 5% (or 10, or 20, or 40%), the durations of the laser pulses should be less than *T*95 = 1.3 fs (or *T*90 = 2.0 fs, or *T*80 = 4.7 fs, or *T*60 = 6.1 fs). For example, ultrafast charge migration in HCCI^+^ is reconstructed in Ref. (Kraus et al., 2015). for the first period of 1.85 fs. By extrapolation of our results, the error of the reported charge migration in HCCI^+^ for the first period is less than 10%. For short pulses with durations up to *T*60, the relative errors of the FNA are found to be almost independent of the amplitudes of the laser pulses. The results of the present work are expected to provide valuable guidance to future investigations of short pulse excitations of HCCI^+^.

## Data Availability

The original contributions presented in the study are included in the article/supplementary material, further inquiries can be directed to the corresponding author.
